# Cystic duplication of lower esophagus in children case report

**DOI:** 10.1016/j.ijscr.2025.111481

**Published:** 2025-06-11

**Authors:** Boumediene Abou-Bekr, Dalila Boumeslout, Nassima Azzouz, Faiza Bouhmama, Yamina Ouadah

**Affiliations:** Department of Pediatric Surgery, Mother and Child Specialty Center at the University AbouBekr Belkaid Faculte of Medecine Tlemcen, Algeria

**Keywords:** Esophagus, Digestive duplication, Child, Duplication cyst

## Abstract

**Introduction:**

Esophageal duplication is a rare form of digestive duplication. This congenital malformation may remain silent or manifest itself by respiratory signs such as compression.

**Case presentation:**

We reported 1 case of esophageal duplication. This was a 12-year-old male child born at term, admitted to our level for chest pain and dysphagia evolving for 4 months. Upon further evaluation, fluid formation at the level of the right lower esophageal latero medial mediastinum was discovered and surgical intervention was necessary. The final diagnosis was an esophageal duplication cyst.

**Discussion:**

Esophageal duplications are rare and represent 15 to 20 % of digestive duplications, often manifesting by respiratory and digestive signs, thus complicating the diagnosis. Imaging, including CT, esophageal transit or fibroscopy, consists of identifying the extent of the formation. Surgery is necessary in all cases before serious complications appear.

**Conclusion:**

The Cystic duplication of lower esophagus are rare and often pose a problem for positive diagnosis. Their treatment is surgical and must be undertaken before the onset of complications.

## Highlights

• Esophageal duplication is a rare form of digestive duplication.
• This is a 12-year-old male child admitted to our level for chest pain and dysphagia lasting 4 months.
• A chest CT scan and an upper digestive endoscopy + esophageal transit are used to make the diagnosis.
• Treatment is surgical and must be undertaken before complications appear.

## 1 Introduction

Esophageal duplications are rare congenital malformations representing 20 % of all digestive duplications. They are the second most common duplication of the digestive tract after that of the ileum. Among these duplications, tubular forms are much rarer than cystic forms. They are most often associated with other congenital malformations, especially vertebral ones [1].

This work has been reported in line with the SCARE criteria [5].

## 2 Observation

**Fig. 1:**
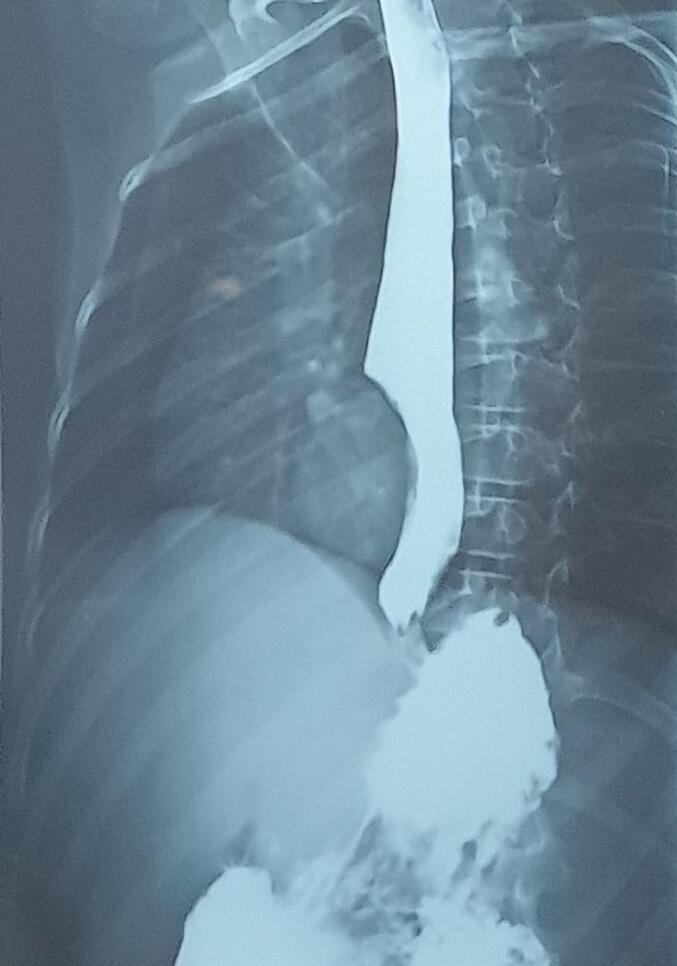
Chest X-ray opaque oval shape in favor of a lower middle mediastinal mass.

**Fig. 2:**
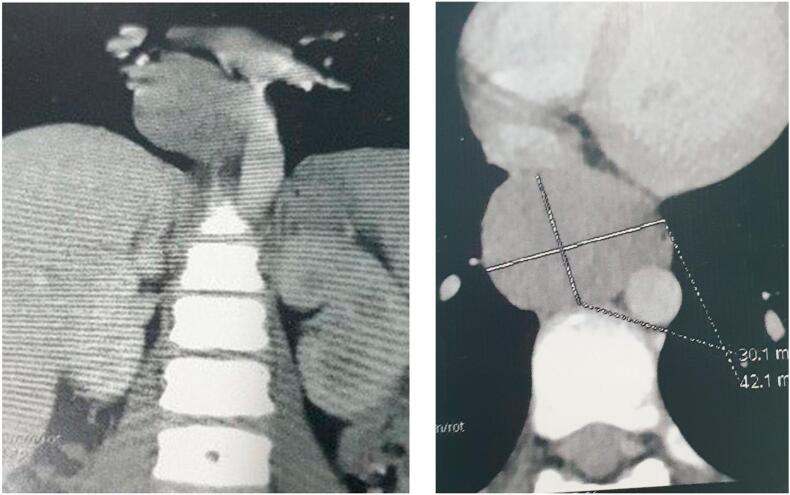
Chest CT scan showing cystic formation of the middle mediastinum and right lower esophagus.

This is a 12-year-old male child, born at term, originally from and residing in Tlemcen, algeria, with no particular medical or surgical history. He was admitted to our level for chest pain and dysphagia for 4 months, with regurgitation, hoarse voice, hepatomegaly and bilateral cervical and axillary adenopathies. A chest x-ray objectifying an oval opacity with an invisible internal limit connecting in a gentle slope to the mediastinum in favor of a lower middle mediastinal mass (Fig. 1). A chest CT scan showing fluid formation in the middle mediastinum and right lower esophagus, without abdominal extension (Fig. 2).

‐ An upper digestive fibroscopy + esophageal transit revealed a rounded formation attached to the right lateral wall of the esophagus, 3–4 cm wide by 4-5 cm up to above the cardia (Fig. 3) (video fibro).
  An upper digestive fibroscopy + esophageal transit revealed a rounded formation attached to the right lateral wall of the esophagus, 3–4 cm wide by 4-5 cm up to above the cardia (Fig. 3) (video fibro).
  **Fig. 3:**
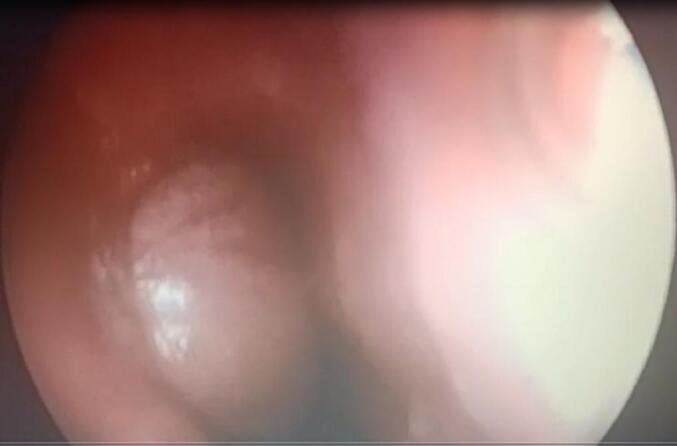
Upper digestive fibroscopy showing a rounded formation attached to the right lateral wall of the esophagus up to above the cardia.

**Fig. 4:**
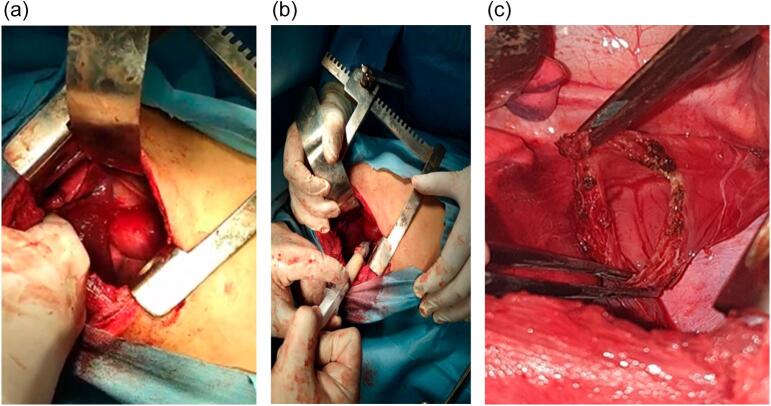
(a) Thoracotomy (b) Cyst puncture; (c) Cyst resection.

The patient received surgical treatment: lateral thoracotomy at the level of the 5th intercostal space and cyst puncture (brought back a whitish liquid). Biopsies: Document sent for an atomopathological study. Result: cystic duplications of the esophagus. (Figs. 4(a), 4(b) and 4(c)).

**Fig. 5:**
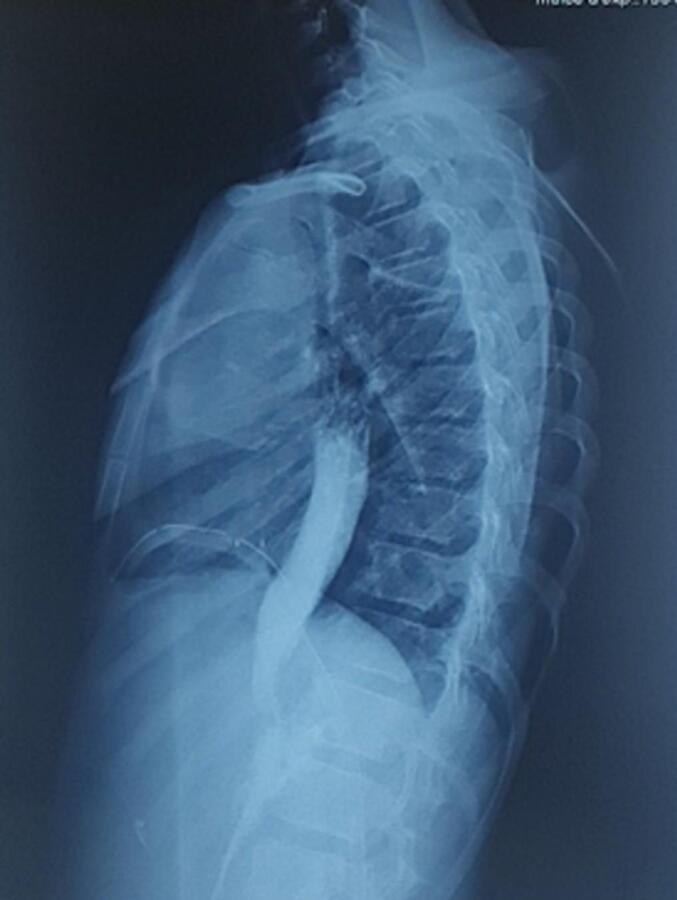
Opaque chest x-ray postoperatively.

During the postoperative phase, the patient experienced no complications. Postoperative imaging findings (opaque chest X-ray) showed no abnormalities. (Fig. 5).

the postoperative follow-up aims to detect complications early and ensure optimal recovery.

1. Immediate Postoperative Monitoring (Early Phase):
  Vital signs: Blood pressure, heart rate, oxygen saturation.
  Pain management: Assessment and appropriate analgesic treatment.
  Nutrition: Parenteral nutrition if needed.
  Gradual reintroduction of oral feeding after confirming esophageal integrity (e.g., barium swallow test*).
  Monitoring for early complications:
  Anastomotic leaks (if esophageal repair was necessary) → fever, tachycardia, chest pain, subcutaneous emphysema.
  Infections (abscess, mediastinitis) → fever, septic signs.
  Respiratory distress (due to postoperative edema or tracheal compression).
2. Medium-Term Monitoring (Hospital Stay & Discharge).
  Radiologic assessment (barium swallow test*) before full oral feeding.
  Prevention of respiratory complications → respiratory physiotherapy if needed.
  Wound care and surgical follow-up.
3. Long-Term Monitoring.
  Esophageal stricture → Watch for dysphagia, endoscopic assessment if needed.
  Recurrence or late complications (fistula, gastroesophageal reflux).
  Normal development (in children) → Growth and feeding assessment.
  The evolution: The patient progressed well without any complications.

## 3 Discussion

### 3.1 Definition and epidemiology

Digestive duplications are rare congenital anomalies, observed in one in 4500 newborns with a M/F sex ratio of 1.2/ 1. The term “digestive duplication” was first used by Ladd in 1941. It corresponds to a set of spherical or tubular cysts, communicating or not, which have in common a contact with any segment of the digestive tract, from the mouth to the anus; a muscular wall with 2 layers of smooth muscle fibers; and a mucosa lined with a digestive epithelium [1]. Their wall comprises a smooth muscularis, made up of 2 layers of different orientation, and a digestive or respiratory type mucosa; which is compatible with the embryological origin of the esophagus. They represent 10 to 20 % of esophageal malformations and 15 to 20 % of digestive duplications [2]. They are discovered in 25 % of cases during the neonatal period, and before the age of 2 years in 70 to 95 % of cases.

### 3.2 Etiology

The exact cause is not fully understood, but several theories explain its origin: [10].

1. Embryological Theories:*Aberrant Foregut Development (Most Accepted Theory): Occurs between the 3rd and 6th week of gestation,Results from incomplete vacuolization and separation of the primitive foregut. *Split Notochord Syndrome:Suggests that defective notochord development affects adjacent foregut structures,Can be associated with spinal anomalies (e.g., vertebral defects). *Incomplete Esophageal Diverticulum Formation:The esophagus normally forms from a single tube, but an incomplete budding process can lead to duplication.
2. Genetic & Environmental Factors:No specific genetic mutations have been identified, but some cases occur with other congenital anomalies (e.g., VACTERL association: Vertebral, Anorectal, Cardiac, Tracheoesophageal, Renal, Limb anomalies).Possible environmental influences during fetal development.

Elsewhere, the duplication may remain asymptomatic and only manifest itself in adulthood (25 % of cases) [1].

There are different forms of esophageal duplication: [1].

‐ Cervical esophageal duplications or tubular thoracic duplications with cervical extension, most often communicating; intradural esophageal duplications, manifested by signs of partial esophageal obstruction, which may or may not be communicating; enteric cysts located in the posterior mediastinum, most often on the right. They are in contact with the esophagus, but do not always share the same wall. Bronchogenic cysts, although of the same origin and similar presentation, are differentiated by the presence of a ciliated mucosa, of the respiratory type, and by the possible presence of cartilaginous tissue [1,4].
‐ Neurenteric cysts which represent a particular form of duplication associated with vertebral anomalies [4] (hemivertebra, segmentation anomaly, partial fusion and body cleft). They are most often dorsal and prevertebral, but are sometimes intraspinal [4]. “Neurenteric cysts” constitute another important subset of esophageal duplications and represent duplications of the esophagus that extend into the spinal canal [9].
‐ Esophageal duplication most often corresponds to a cystic structure in the right laterotracheal position. The tubular form remains extremely rare [1].

### 3.3 The diagnosis of esophageal duplication

#### 3.3.1 Clinical signs

Symptoms of esophageal duplication usually appear early in life, with most patients presenting before 2 years of age. The presentation of esophageal duplication depends on four factors [8]:

(1) the anatomic level/location of the lesion; (2) mass effect of the lesion; (3) complications secondary to luminal secretions; and (4) cyst infection. The majority of esophageal duplications are discovered incidentally in asymptomatic patients. When symptomatic, the most common presentation is respiratory symptoms secondary to airway compression/mass effect [8]. Therefore, it has been suggested that duplication should be considered in the differential diagnosis of all cases of respiratory obstruction in the newborn. Other less common symptoms are considered to be due to the presence of ectopic gastric tissue and may include pain (secondary to inflammation or rupture), distension, hemoptysis, and peptic ulceration secondary to atopic gastric mucosa (sometimes leading to gastrointestinal hemorrhage) [9].

#### 3.3.2 Imaging studies

Identification of the duplication cyst is usually made by chest radiograph obtained either secondary to respiratory symptoms or incidentally. AP and lateral chest radiographs can demonstrate a round, mid- or posterior mediastinal opacity of water tone. Signs of direct compression on the trachea or a main bronchus should be looked for [4]. There may also be indirect signs of compression (obstructive emphysema, atelectasis) or a mobile air-fluid level with change of position if the duplication is communicating [1]. Posteroanterior and lateral radiographs have been shown to detect more than 90 % of lesions [8]. It is important that children with spinal abnormalities or suspected neuroenteric cysts undergo MRI to determine the extent, if any, of any spinal involvement prior to surgical treatment [9].

‐ The esophageal transit may be normal or show extrinsic compression in cystic forms, the importance of which depends on the volume of the duplication and its degree of contact with the esophagus, the latter being able to be displaced by the mass or the latter being able to be visible in the form of an intradural and extra mucosal lesion, with a widening of the lumen on both sides [4]. In tubular forms generally communicating, it allows to easily visualize the duplication [1].
‐ -Tc-99m pertechnetate scintigraphy can be used to identify ectopic gastric mucosa in these cysts [8].
‐ -Magnetic resonance imaging (MRI) may be helpful to better delineate anatomic relationships and exclude other abnormalities, such as GISTs or leiomyomas. Esophageal duplication cysts have high signal intensity on T2-weighted images because of the high proportion of water in the cyst contents. [8].

### 3.4 Differential diagnosis

The differential diagnosis of esophageal duplication include [11]: Bronchogenic cyst – May appear similar on imaging but often located near the tracheobronchial tree.Esophageal cyst (enteric cyst): A broader category that includes duplication cysts. Lymphatic malformation (lymphangioma): Can present as a cystic mass in the mediastinum. Neuroenteric cyst –:Typically associated with vertebral anomalies.Teratoma or dermoid cyst: May contain calcifications and fat. Mediastinal abscess – Can mimic a cystic lesion but is often associated with infection signs. Congenital pulmonary airway malformation (CPAM):If located near the esophagus. Thoracic meningoceles:In cases of posterior mediastinal cysts.

Imaging (MRI, CT, or endoscopic ultrasound) and histopathology help differentiate these entities.

The differential diagnosis is discussed especially in the face of posterior mediastinal masses, in particular bronchogenic cysts and neurogenic tumors. Thus, for bronchogenic cysts, CT scans can differentiate by visualizing the cartilage in the cyst wall. For neurogenic tumors, ultrasound and CT scans highlight the solid nature of the lesion. Sometimes, the diagnosis can be difficult and is then only confirmed by histological study [1].

## 4 Treatment

Surgical planning is performed based on the characteristics of each duplication. The approach is initially dictated by the level of the lesion: cervical, thoracic or thoracoabdominal [1]. Complete excision of the duplication remains the norm [3,5] by two methods: thoracotomy [3,6] and video-assisted thoracoscopic surgery [6,8].

The approach is adapted to the location of the cyst: cervicotomy; right or left lateral postero-thoracotomy.

The complications associated with esophageal cysts include heartburn, reflux esophagitis, rupture, obstruction, hemorrhage, infection, and malignancy. Other complications related to esophageal cyst intervention include tracheal and esophageal injuries, pseudodiverticulum development, and nerve injury/paralysis. [10]

Long-term outcome is favorable in the case of total excision [7]. Preservation of part of the mucosa exposes to the risks of mucoid effusion, hemothorax, digestive hemorrhage and perforation in the case of gastric heterotopia [2]. Carcinomatous degeneration is exceptional. Ten cases of digestive duplication degeneration have been reported, of which only two concerned the esophageal location [8]. The age of presentation varied widely from 18 to 60 years, with no gender predominance and variable size from 3 to 10 cm. The clinical presentation of these malignant tumors ranged from incidental discovery to dysphagia, fever and pain. [8].

## 5 Conclusion

The Cystic duplication of lower esophagus is a rare congenital malformation. The clinical symptomatology is polymorphic and depends on the type, location and size of the duplicity. It remains dominated by respiratory and digestive signs.

The positive diagnosis is essentially radiological. The search for other associated digestive and especially vertebral anomalies is essential. Their treatment is surgical and must be undertaken before complications appear.

The following are the supplementary data related to this article.

Supplementary video Supplementary video

## Author contribution

**Boumediene Abou-Bekr**: study concept.

**Dalila Boumeslout**: data collection data analysis.

**Nassima Azzouz**: contributors.

**Faiza Bouhmama**: data collection.

**Yamina Ouadah**: writing the paper.

## Consent

Consent statement for a minor: Written informed consent was obtained from the patient's parents/legal guardian for publication and any accompanying images. A copy of the written consent is available for review by the Editor-in-Chief of this journal on request.

## Ethical approval

This study did not require ethical approval as it is a case report. However, informed consent has been obtained and can be provided on request.

## Guarantor

Bomediene Abou-Bekr.

## Sources of funding

This research did not receive any specific grant from funding agencies in the public, commercial, or not for-profit sectors.

## Declaration of competing interest

Authors declare no conflict of interest.
